# Supplementary material for: MetaRibo-Seq measures translation in microbiomes
Source: Nat Commun. 2020 Jun 29;11:3268. doi: 10.1038/s41467-020-17081-z (PMC7324362; doi:10.1038/s41467-020-17081-z)
Supplement: Supplementary file 10 — Supplementary Data 7 [file 41467_2020_17081_MOESM10_ESM.zip › File2/Confidence_High_Taxonomy/345891_out.krona.html]

Javascript must be enabled to view this page.

members
magnitude
magnitudeUnassigned
count
unassigned
taxon
rank

345891\_out

56

superkingdom
2
56

56
1239
phylum

class
186801
56

1

SRS023914\_contig\_number\_16985
186802
order
56

family
26
186803
7

SRS012273\_contig\_number\_contig-100\_1056.251523SRS015854\_contig\_number\_8368SRS016495\_contig\_number\_20098SRS044535\_contig\_number\_8758SRS050998\_contig\_number\_11421SRS062654\_contig\_number\_25132SRS063813\_contig\_number\_111

1506553
genus
1

1
1871021
species

SRS104485\_contig\_number\_4923


SRS050925\_contig\_number\_26057
1
species
39491

genus
841
1

1
1638781
species

SRS143181\_contig\_number\_14669

genus
189330
12

1
2292347
species

SRS017433\_contig\_number\_10715


SRS018427\_contig\_number\_contig-100\_9.191489SRS018623\_contig\_number\_16430SRS077502\_contig\_number\_19314SRS1041033\_contig\_number\_13816SRS893230\_contig\_number\_7717SRS893270\_contig\_number\_16215SRS971275\_contig\_number\_15677
39486
species
7


SRS020328\_contig\_number\_38442SRS024009\_contig\_number\_10647SRS078419\_contig\_number\_37387SRS143991\_contig\_number\_contig-100\_144.169196
88431
species
4

2302965
species
4

SRS057478\_contig\_number\_7009SRS063190\_contig\_number\_12691SRS1055076\_contig\_number\_17200SRS144183\_contig\_number\_42449

25
family
541000

genus
1263
25

24
165186
species

SRS015264\_contig\_number\_5501SRS016335\_contig\_number\_16499SRS016989\_contig\_number\_12696SRS019685\_contig\_number\_41291SRS024132\_contig\_number\_contig-100\_1019.279274SRS024492\_contig\_number\_contig-100\_40.121030SRS043701\_contig\_number\_7432SRS043841\_contig\_number\_1777SRS048164\_contig\_number\_19213SRS048870\_contig\_number\_contig-100\_102.107913SRS049402\_contig\_number\_contig-100\_85.75969SRS050422\_contig\_number\_17637SRS052697\_contig\_number\_45238SRS058070\_contig\_number\_contig-100\_433.164463SRS063040\_contig\_number\_30215SRS063985\_contig\_number\_11261SRS077194\_contig\_number\_32885SRS097920\_contig\_number\_24847SRS103987\_contig\_number\_24133SRS142503\_contig\_number\_58209SRS146812\_contig\_number\_60635SRS148196\_contig\_number\_37871SRS148721\_contig\_number\_56386SRS149181\_contig\_number\_26082


SRS015890\_contig\_number\_15772
1
species
46228

4
family
186806

1730
genus
4


SRS015578\_contig\_number\_17694SRS1041144\_contig\_number\_3335
2
1262889
species


SRS018656\_contig\_number\_9924SRS143876\_contig\_number\_37802
2
39496
species
